# Chemical Constituents from the Flower of *Hosta plantaginea* with Cyclooxygenases Inhibition and Antioxidant Activities and Their Chemotaxonomic Significance

**DOI:** 10.3390/molecules22111825

**Published:** 2017-10-26

**Authors:** Li Yang, Shu-Tai Jiang, Qin-Guang Zhou, Guo-Yue Zhong, Jun-Wei He

**Affiliations:** 1 Key Laboratory of Modern Preparation of TCM, Jiangxi University of Traditional Chinese Medicine, Ministry of Education, Nanchang 330004, China; yangli07971@163.com; 2 Institute of Traditional Chinese Medicine and Natural Products, College of Pharmacy, Jinan University, Guangzhou 510632, China; vstjiang@stu2014.jnu.edu.cn; 3 Research Center of Natural Resources of Chinese Medicinal Materials and Ethnic Medicine, Jiangxi University of Traditional Chinese Medicine, Nanchang 330004, China; qingguangzhou58@gmail.com (Q.-G.Z.); zgy1037@163.com (G.-Y.Z.)

**Keywords:** *Hosta plantaginea*, chemical constituents, cyclooxygenase inhibition, antioxidant, chemotaxonomics

## Abstract

Two new phenolic glucosides, hostaflavanone A (**1**) and *anti*-1-phenylpropane-1,2-diol-2-*O*-β-d-glucopyranoside (**2**), together with six known compounds, *anti*-1-phenylpropane-1,2-diol (**3**), phenethyl-*O*-β-d-glucopyranoside (**4**), phenethanol-β-d-gentiobioside (**5**), phenethyl-*O*-rutinoside (**6**), (1*S*, 3*S*)-1-methyl-1,2,3,4-tetrahydro-β-carboline-3-carboxylic acid (**7**), and (1*R*, 3*S*)-1-methyl-1,2,3,4-tetrahydro-β-carboline-3-carboxylic acid (**8**), were isolated from the flower of *Hosta plantaginea*, and their structures were elucidated by nuclear magnetic resonance (NMR), high resolution electrospray ionization mass spectroscopy (HRESIMS), and circular dichroism (CD) analyses. The cyclooxygenases (COX-1 and COX-2) inhibition and antioxidant activities of compounds **1** and **4**–**6** were investigated, and they showed moderate cyclooxygenases inhibition activities. Moreover, only compound **1** exhibited moderate antioxidant activity, with an IC_50_ value of 83.2 μM, while **4**–**6** showed insignificant activity with IC_50_ values of 282, 257, and 275 μM, respectively. This is the first report of compounds **3** and **5**–**8** from the Liliaceae family. The chemotaxonomic significance of the isolated compounds was also summarized.

## 1. Introduction

**Figure 1:**
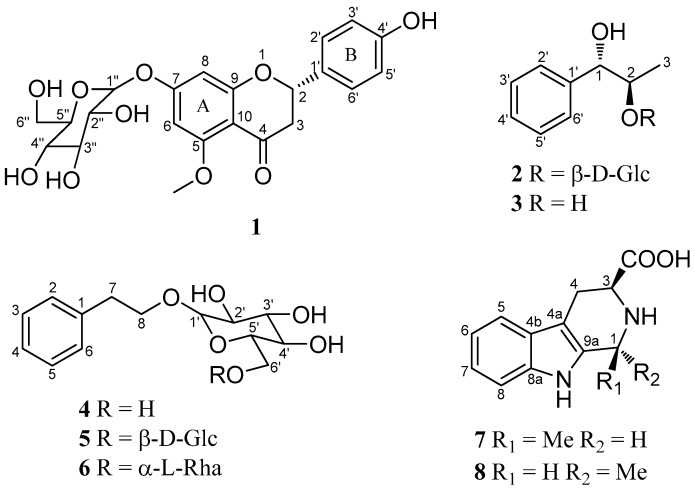
Chemical structures of compounds **1**–**8**.

The genus *Hosta* belongs to the family Liliaceae, with approximately 40 species distributed in the temperate and subtropical zones of Asia [1]. The ethnopharmacological and chemotaxonomic significance of the genus *Hosta* led us to investigate the chemical constituents of one of its species, namely *Hosta plantaginea* (Lam.) Aschers, which was a medicinal and ornamental plant in China. Its flowers are commonly used as a traditional Mongolian medicine in China for the treatment of sore throat, mute, lung heat, and toxic heat [2]. Previous phytochemical studies on *H. plantaginea* afforded structurally-diverse and biologically-active compounds, such as steroidals, alkaloids, flavonoids, and monoterpenes, some of them showed potent anti-inflammatory, cytotoxic, antibacterial, antiviral, and antioxidant activities [3,4,5,6,7,8]. These facts encouraged us to investigate new and bioactive secondary metabolites from *H. plantaginea*. In the present study, we had isolated and elucidated two new phenolic glucosides (**1** and **2**), and six known ones from the ethanol extract of the flowers of *H. plantaginea*. Herein, we report the isolation, structure elucidation, as well as the cyclooxygenases’ (COX-1 and COX-2) inhibition and antioxidant activities of compounds **1**–**8** (Figure 1). This is the first report of compounds **3** and **5**–**8** from the Liliaceae family. The chemotaxonomic significance of the isolated compounds was also summarized.

## 2. Results and Discussion

### 2.1. Identification of Compounds ***1***–***8***

**Figure 2:**
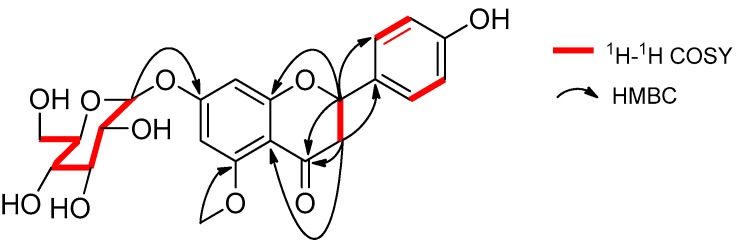
Selected ^1^H-^1^H COSY and HMBC correlations of **1**.

**Figure 3:**
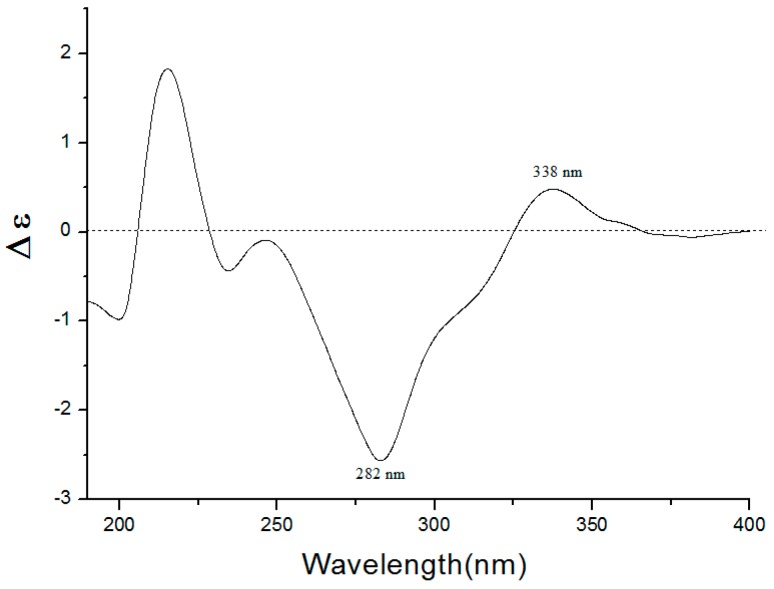
CD spectrum of compound **1** in CH_3_OH.

Compound **1** was isolated as a yellow oil, ${\left[\alpha \right]}_{D}^{25}$ −48.2 (*c* 1.0, CH_3_OH), had a molecular formula of C_22_H_24_O_10_ on the basis of the HR-ESI-MS (*m*/*z* 449.14498, calcd. 449.14422 [M + H]^+^). The UV absorption bands at λ_max_ 281 and 322 nm suggested the presence of a flavanone skeleton in **1** [9]. The ^1^H-NMR spectrum of **1** (Table 1, see the Supplementary Materials) exhibited a pair of meta-positioned aromatic protons at *δ*_H_ 6.28 and 6.24 (each, 1H, d, *J* = 2.5 Hz) in ring A, an AA’XX’ coupling system at *δ*_H_ 7.30 and 6.79 (each, 2H, d, *J* = 8.4 Hz) in ring B. The ^13^C-NMR spectrum of **1** (Table 1) combined with DEPT 135 spectrum displayed 22 resonances for a carbonyl carbon (*δ*_C_ 188.3), 12 aromatic carbons (*δ*_C_ 164.1, 163.3, 161.7, 157.7, 129.1, 128.3, 128.3, 115.2, 115.2, 106.1, 96.1, and 94.0), one oxymethine carbon (*δ*_C_ 78.3), one methoxyl group (*δ*_C_ 55.9), one methylene carbon (*δ*_C_ 44.7), and a d-glucosyl moiety (*δ*_C_ 99.7, 77.2, 76.5, 73.1, 69.7, and 60.7), which was supported by the result of the acid hydrolysis and HPLC analysis. Based on the above evidence, the aglycone of **1** was identified as flavanone. Additionally, the configuration of the anomeric carbon was deduced to be *β* based on the coupling constant of the anomeric proton (H-1′′ 7.8 Hz). The glucosidic linkage was established by the HMBC correlation (Figure 2) between H-1′′ (*δ*_H_ 4.99) and C-7 (*δ*_C_ 163.3), indicating that the glucosyl moiety was attached to C-7. Moreover, the methoxyl group was located at C-5 by the HMBC correlation from 5-OCH_3_ (*δ*_H_ 3.78) to C-5 (*δ*_C_ 161.7). With the aid of ^1^H-^1^H COSY, HSQC, and HMBC correlations allowed the established the planar structure and assigned all the ^1^H- and ^13^C-NMR signals of **1**, which was isolated from *Prunus cerasoides* and named puddumin A [10]. However, only incomplete 1D-NMR data of **1** was given in the literature 10, which was also differ greatly from our data (Table 1). The electronic circular dichroism (CD) spectrum of **1** (Figure 3) showed a negative Cotton effect at 282 nm (π→π^*^ electronic transition) and a positive Cotton effect at 338 nm (n→π^*^ electronic transition), suggesting that the absolute configuration at C-2 was *S* [9,11]. Thus, the structure of **1** was fully elucidated, and it was named hostaflavanone A.

Compound **2** was isolated as a yellow oil, had a molecular formula of C_15_H_22_O_7_ on the basis of the HR-ESI-MS (*m/z* 315.14283, calcd. 315.14383 [M + H]^+^). The UV absorption bands at λ_max_ 224 and 274 nm. The ^1^H-NMR spectrum of **2** (Table 2) exhibited an AAʹXXʹ coupling system at *δ*_H_ 7.16–7.26 (5H, m), one methxyl at *δ*_H_ 1.16 (3H, d, *J* = 6.3 Hz). The ^13^C-NMR spectrum of **2** (Table 2) displayed 15 resonances for six aromatic carbons (*δ*_C_ 140.1, 129.3, 129.3, 127.9, 127.9, and 125.6), two oxymethine carbons (*δ*_C_ 78.6 and 73.7), one methxyl group (*δ*_C_ 16.5), and a glucosyl moiety (*δ*_C_ 102.9, 76.8, 76.4, 73.5, 70.1, and 61.0). The ^1^H and ^13^C-NMR spectra were similar to those of the known compound **3** [12], expect for the presence an additional glucosyl moiety signals. The major differences in the chemical shifts for C-1 (ΔC +2.5), C-2 (ΔC +4.3), and C-3 (ΔC -2.6) were ascribed to glycosylation, suggesting that the glucosyl moiety was located at C-2. Moreover, the configuration of the anomeric carbon was deduced to be *β* based on the coupling constant of the anomeric proton (H-1′′, 7.8 Hz). The small coupling constant ^3^*J*_1,2_ (5.1 Hz) of **2** and **3** (Table 2) indicated that **2** and **3** were in the *anti*-configuration. According to the literatures, the *anti*-phenylpropane-1,2-diol displayed smaller coupling constants of ^3^*J*_1,2_ (4.4–5.4 Hz) than *syn*-phenylpropane-1,2-diol (^3^*J*_1,2_ 7–8 Hz) [12,13,14,15,16,17,18]. Due to the shortage of the sample, the two-dimensional (2D)-NMR experiments were not carried out. On the basis of the above evidence, the planar structure of **2** was deduced as *anti*-1-phenylpropane-1,2-diol-2-*O*-β-d-glucopyranoside.

By comparison of the NMR and MS data with those reported, compounds **3**–**8** isolated from the flower of *H. plantaginea* were identified as *anti*-1-phenylpropane-1,2-diol (**3**) [12], phenethyl-*O*-β-d-glucopyranoside (**4**) [19], phenethanol-β-gentiobioside (**5**) [20], phenethyl-*O*-rutinoside (**6**) [21], (1*S*,3*S*)-1-methyl-1,2,3,4-tetrahydro-β-carboline-3-carboxylic acid (**7**) [22], and (1*R*,3*S*)-1-methyl-1,2,3,4-tetrahydro-β-carboline-3-carboxylic acid (**8**) [22], respectively.

### 2.2. Biological Activities

Compounds **1** and **4**–**6** exhibited moderate activity to that of the standard reference drug, and were tested for their inhibitory activity against ovine COX-1 and COX-2 (Table 3), with IC_50_ values of 15.5–41.2 and 31.7–45.4 μM, while the IC_50_ values of the positive control celecoxib were 9.0 and 1.0 μM, respectively. While compounds **1** and **4** were more active against COX 1 (SI values <<1), compounds **5** and **6** were about equally potent against both COX enzymes with SI values of about 1.

The antioxidant activity of compounds **1**, and **4**–**6** was measured by the 1,1-diphenyl-2-picrylhydrazyl (DPPH) method and the results are summarized in Table 3. Only compound **1** exhibited moderate antioxidant activity, with an IC_50_ value of 83.2 μM, while **4**–**6** showed insignificant activity with IC_50_ values of 282, 257, and 275 μM, respectively. The IC_50_ value of the positive control l-ascorbic acid was 33.9 μM. These compounds may thus, possibly together with further constituents, contribute to the biological activity of *H. plantaginea*.

### 2.3. Chemotaxonomic Significance

In our present study, eight compounds including one flavanone (**1**), two phenylpropanoids (**2** and **3**), three phenethanols (**4**–**6**), and two β-carboline alkaloids (**7** and **8**) were isolated from the flowers of *H. plantaginea*. Hostaflavanone A (**1**) and *anti*-1-phenylpropane-1,2-diol-2-*O*-β-d-glucopyranoside (**2**) were identified as two new ones, and this is the first report of compounds **3**, and **5**–**8** from the Liliaceae family. Additionally, the structure types of flavanone and β-carboline alkaloid from Liliaceae family for the first time.

The phenylpropanoids have been previously isolated from the *Hosta* species, including *trans*-*p*-hydroxy-cinnamic acid from *H. ventricosa* [23], *p*-coumaramide, *trans*-*N*-*p*-coumaroyltyramine, and *cis*-*N*-coumaroyltyramine from *H. longipes* [24], feruloyltyramine, and lyciumide A from *H. ensata* [25]. In addition, the phenethanols **4** and α-hydroxyacetovanillone were isolated from *H. plantaginea* and *H. ventricosa* [23], respectively. Thus, compounds **2**–**6** from *H. plantaginea*, suggesting that their occurrence could be used to verify the chemotaxonomic relationship of *H. plantaginea* and other species of *Hosta*, and also might sever as valuable chemotaxonomic makers for the identification of *H. plantaginea*. Further comprehensive phytochemical investigations involving an expand series of compounds could help define the chemotaxonomic significance of species belonging to genus *Hosta*.

## 3. Experimental Section

### 3.1. General Procedures

Optical rotations were measured using a JASCO P-1020 polarimeter (JASCO Corporation, Tokyo, Japan). The UV spectra were recorded in CH_3_OH using a JASCO V-550 UV-VIS spectrophotometer (JASCO Corporation, Tokyo, Japan). ^1^H (600 MHz), ^13^C (150 MHz), DEPT 135 (150 MHz), and 2D (^1^H-^1^H COSY, HSQC, and HMBC) NMR spectra were recorded on a Bruker AV 600 spectrometer (Bruker Corporation, Fallanden, Switzerland). HR-ESI-MS was measured on a Waters Synapt G2 TOF mass spectrometer (Waters Corporation, Manchester, UK). Column chromatographies (CCs) were carried out on silica gel (200–300 mesh, Marine Chemical Group Corporation, Qingdao, China) and ODS (60–80 µm, YMC, Tokyo, Japan). Silica gel GF_254_ (Marine Chemical Group Corporation, Qingdao, China) was used for analytical TLC. 2,2-Di-phenyl-1-picrylhydrazyl (DPPH) (Sigma Corporation, Ronkonkoma, New York, NY, USA), l-cysteine methyl ester and *o*-tolyl isothiocyanate (Meilun Biotech. Co. Ltd., Dalian, China), d-glucose, and l-glucose (Energy Chemical, Shanghai, China) were used. COX inhibitor screening assay kit was purchased from Cayman Chemical Company (Ann Arbor, MI, USA). The analytical HPLC was performed on a Shimadzu HPLC system equipped with an LC-20AB pump, and a SPD-20A diode array detector (Shimadzu, Kyoto, Japan), using a Phenomenex Gemini C18 column (5 μm, 4.6 mm × 250 mm, Phenomenex Inc., Los Angeles, CA, USA). The preparative HPLC was performed on a Shimadzu LC-6AD system equipped with an LC-6AD pump and an SPD-M20A detector (Shimadzu, Kyoto, Japan), using an RP-18 column (5 μm, 21.2 × 250 mm, Gemini, Phenomenex Inc., Los Angeles, CA, USA; detector set at 220 and 254 nm).

### 3.2. Plant Materials

The flowers of *Hosta plantaginea* (Lam.) Aschers were collected in Shanquan town, Nanchuan district, Chongqing, People’s Republic of China, in September 2014, and were identified by one of authors (Guo-yue Zhong). A voucher specimen (no. YZH201409) was deposited at the Research Center of Natural Resources of Chinese Medicinal Materials and Ethnic Medicine, Jiangxi University of Traditional Chinese Medicine, Nanchang, China.

### 3.3. Extraction and Isolation

The air-dried and powdered flowers of *H. plantaginea* (16.5 kg) were extracted three times with 80% EtOH (40 L) by maceration at room temperature for three days. After filtration, combination, and solvent evaporation the residue (6.60 kg) was dissolved in water and successively partitioned with petroleum ether, ethyl acetate, and *n*-BuOH to afford petroleum ether (A, 363 g), ethyl acetate (B, 127 g), *n*-BuOH (C, 804 g), and water (5.27 kg) extracts, respectively. The *n*-BuOH extract (760 g) was subjected to HP20 macroporous adsorption resin column chromatography (CC) eluting with H_2_O, 20%, 50%, and 95% aqueous EtOH to give four fractions, c1, c2, c3, and c4, respectively. Fr. c2 (32.8 g) was subjected to MCI CC using a EtOH/H_2_O gradient elution (10%, 20%, 30%, and 95%) to give four fractions (c21 to c2d). The subfraction c2c (6.02 g) was applied to silica gel CC eluting with dichloromethane–CH_3_OH (5:1, 3:1, 1;1, 0:100, *v*/*v*) to afford four subfractions (c2c1–c2c4). c2c2 (1.04 g) was purified by pre-HPLC eluting with CH_3_OH/H_2_O (*v*/*v*, 35:65, flow rate: 10 mL/min) to afford compound **1** (53.7 mg, t_R_ 22.7 min). The subfraction c2b (15.1 g) was applied to polyamide CC using a EtOH/H_2_O gradient elution (10%, 20%, and 95%) to afford five subfractions (c2b1–c2b5). The subfraction c2b1 (7.12 g) was applied to silica gel CC eluting with dichloromethane–CH_3_OH (10:1, 5:1, 1:5, *v*/*v*) to afford four subfractions (c2b11–c2b14). c2b11 (1.81 g) was purified by pre-HPLC eluting with CH_3_CN/H_2_O (*v*/*v*, 20:80, flow rate: 2 mL/min) to afford compounds **2** (1.0 mg, t_R_ 15.0 min), **3** (0.7 mg, t_R_ 25.3 min), and **4** (1.45 g, t_R_ 17.2 min). c2b12 (1.19 g) was purified by pre-HPLC eluting with CH_3_OH/H_2_O (*v*/*v*, 30:70, flow rate: 10 mL/min) to afford compound6 **6** (1.10 g, t_R_ 31.0 min). The subfraction c2a (8.05 g) was applied to silica gel CC eluting with dichloromethane/CH_3_OH (1:1, 1:3, 1:10, *v*/*v*) to afford three subfractions (c2a1–c2a3). c2a1 (3.89 g) was subjected to Sephadex LH-20 CC eluting with CH_3_OH to afford three subfractions (c2a11–c2a13). c2a12 (2.59 g) was purified by pre-HPLC eluting with CH_3_OH/H_2_O (*v*/*v*, 25:75, flow rate: 10 mL/min) to afford compounds **5** (329 mg, t_R_ 32.8 min), **7** (1.9 mg, t_R_ 23.2 min), and **8** (1.4 mg, t_R_ 27.3 min).

### 3.4. Acid Hydrolysis and HPLC Analysis

The absolute configurations of the sugar moieties in the structures were determined by the previously described method with minor modifications [9]. Compound **1** (3 mg) was hydrolyzed with 2 mL of 2 M HCl for 3 h at 90 °C. The mixture was evaporated to dryness in vacuo, and the residue was dissolved in H_2_O and extracted with CHCl_3_. After the aqueous layer was dried in vacuo, the residue was dissolved in pyridine (1 mL) containing l-cysteine methyl ester (1 mg) and heated at 60 °C for 1 h. *o*-Tolyl isothiocyanate (5 μL) was added, and the mixture was heated at 60 °C for 1 h and directly analyzed by HPLC. Analytical HPLC was performed on a reversed-phase C18 column (5 μm, 4.60 × 250 mm; Intertsutain, Shimadzu) at 30 °C with isocratic elution using 25% CH_3_CN containing 0.1% formic acid for 40 min at a flow rate 0.8 mL/min. The peaks were detected with a UV detector at 250 nm. The standard monosaccharides, d-glucose, and l-glucose, were subjected to the same process.

### 3.5. In Vitro COX-1 and COX-2 Inhibitory Assay

Inhibitory activities of the compounds towards COX-1 and COX-2 activity was determined using colorimetric COX (ovine) inhibitor screening assay kit (Cayman, no. 760111) following the manufacturer’s instructions, using celecoxib as a positive control [26]. The 50% inhibitory concentration (IC_50_) values were calculated from the concentration-inhibition response curve.

### 3.6. Antioxidant Assay

DPPH radical-scavenging activity of the sample was measured as previously described with minor modifications [27]. In a 96-well microplate, 150 μL of DPPH solution (200 μM) was added to 50 μL of the test sample in methanol at different concentrations. The OD values of the reaction mixtures was recorded at 517 nm using a Multiskan Go (Thermo Fisher Scientific, Inc., Waltham, MA, USA) for 40 min at 30 °C. The DPPH radical scavenging activity was calculated by the following equation: DPPH scavenging activity % = (A_sample_ – A_blank_)/A_control_ × 100, where A_sample_ represents the absorbance of sample and DPPH, A_blank_ represents the absorbance of sample and CH_3_OH, A_control_ represents the absorbance of DPPH and CH_3_OH. IC_50_ value was calculated as the concentration required to scavenge 50% DPPH free radicals and was obtained by plotting the DPPH-scavenging percentage of each sample against the sample concentration. l-ascorbic acid was used as the positive control in this experiment. All tests were run in triplicate, and values obtained from experiments were averaged.

## 4. Conclusions

In summary, one new flavanone (**1**) and one new phenylpropanoid (**2**), together with six known ones, one phenylpropanoid (**3**), three phenethanols (**4**–**6**), and two β-carboline alkaloids (**7** and **8**), were isolated from the flowers of *H. plantaginea*. This is the first report of compounds **3** and **5**–**8** from the Liliaceae family. Additionally, the structure types of flavanone and β-carboline alkaloid from the Liliaceae family for the first time. Moreover, compounds **2**–**6** from *H. plantaginea* suggest that their occurrence could be used to verify the chemotaxonomic relationship of *H. plantaginea* and other species of *Hosta*, and also might serve as valuable chemotaxonomic makers for the identification of *H. plantaginea*. The cyclooxygenases’ (COX-1 and COX-2) inhibition and antioxidant activities of compounds **1** and **4**–**6** were investigated, and they showed moderate cyclooxygenase inhibition activities. Moreover, only compound **1** exhibited moderate antioxidant activity, with an IC_50_ value of 83.2 μM, while **4**–**6** showed insignificant activity with IC_50_ values of 282, 257, and 275 μM, respectively. These compounds may, possibly together with further constituents, contribute to the biological activity of *H. plantaginea*.

## Figures and Tables

**Table 1:** ^13^C- and ^1^H-NMR data for compounds **1** and puddumin A in DMSO-*d*_6_ (*δ* in ppm).

Position	1		Puddumin A	
	*δ*_C_	*δ*_H_ (*J* in Hz)	*δ*_C_	*δ*_H_ (*J* in Hz)
2	78.3	5.40 (1H, m)	78.3	6.15 (1H, s)
3	44.7	3.07 (1H, dd, *J* = 16.3/12.8)	44.5	3.30 (1H, d, *J* = 2)
		2.57 (1H, dd, *J* = 16.3/2.5)		2.70 (1H, s)
4	188.3	- ^a^	192.4	- ^a^
5	161.7	- ^a^	159.9	- ^a^
6	94.0	6.28 (1H, d, *J* = 2.5)	93.7	6.40 (1H, d, *J* = 2)
7	163.3	- ^a^	165.6	- ^a^
8	96.1	6.24 (1H, d, *J* = 2.5)	95.1	6.78 (1H, d, *J* = 2)
9	164.1	- ^a^	165.1	- ^a^
10	106.1	- ^a^	106.5	- ^a^
1′	129.1	- ^a^	126.0	- ^a^
2′, 6′	128.3	7.30 (2H, d, *J* = 8.4)	130.8	7.65 (2H, d, *J* = 9)
3′, 5′	115.2	6.79 (2H, d, *J* = 8.4)	115.9	6.83 (2H, d, *J* = 9)
4′	157.7	- ^a^	159.6	- ^a^
1′′	99.7	4.99 (1H, d, *J* = 7.8)	100.3	- ^b^
2′′	73.1	3.23 (1H, m)	73.3	- ^b^
3′′	76.5	3.28 (1H, m)	76.8	- ^b^
4′′	69.7	3.14 (1H, m)	69.6	- ^b^
5˝	77.2	3.39 (1H, m)	77.5	- ^b^
6′′	60.7	3.68 (1H, m)	60.6	
		3.43 (1H, m)		^b^
5-OCH_3_	55.9	3.78 (3H, s)	55.5	3.80 (3H, s)

^a^ no signal; ^b^ not assigned.

**Table 2:** ^13^C- and ^1^H-NMR data for compounds **2** and **3** in DMSO-*d*_6_ (*δ* in ppm).

Position	3		2	
	*δ*_C_	*δ*_H_ (*J* in Hz)	*δ*_C_	*δ*_H_ (*J* in Hz)
1	76.1	4.48 (1H, d, *J* = 5.1)	78.6	4.43 (1H, d, *J* = 5.1)
2	69.4	4.41 (1H, m)	73.7	4.40 (1H, m)
3	19.1	1.08 (3H, d, *J* = 6.0)	16.5	1.16 (3H, d, *J* = 6.3)
1′	140.3		140.1	
2′, 6′	127.8	7.15–7.26 (5H, m)	127.9	7.16-7.26 (5H, m)
3′, 5′	129.3	7.15–7.26 (5H, m)	129.3	7.16-7.26 (5H, m)
4′	125.5	7.15–7.26 (5H, m)	125.6	7.16-7.26 (5H, m)
1′′	- ^b^	- ^b^	102.9	4.23 (1H, d, *J* = 7.8)
2′′	- ^b^	- ^b^	73.5	- ^a^
3′′	- ^b^	- ^b^	76.4	- ^a^
4′′	- ^b^	- ^b^	70.1	- ^a^
5′′	- ^b^	- ^b^	76.8	- ^a^
6′′	- ^b^	- ^b^	61.0	- ^a^

^a^ not assigned; ^b^ no signal.

**Table 3:** In vitro COX-1/COX-2 inhibition and antioxidant activities of isolated compounds.

Compounds	IC_50_ (μM)			SI ^d^
	COX-1 ^a^	COX-2 ^a^	Antioxidant ^b^	
**1**	21.6 ± 1.2	45.4 ± 3.3	83.2 ± 3.0	0.48
**4**	15.5 ± 0.6	38.2 ± 3.9	282 ± 5.2	0.41
**5**	38.4 ± 1.3	31.7 ± 2.9	257 ± 13.8	1.16
**6**	41.2 ± 1.5	35.4 ± 1.6	275 ± 14.8	1.21
celecoxib	9.0 ± 0.6	1.0 ± 0.1	- ^c^	9.00
l-ascorbic acid	- ^c^	- ^c^	33.9 ± 1.1	- ^c^

^a^ Meaning the 50% inhibition concentration of isolated compounds calculated from regression using five different concentrations (100, 50, 25, 12.5, 6.25 μM); ^b^ Meaning the 50% inhibition concentration of isolated compounds calculated from regression using five different concentrations (500, 200, 100, 50, 25 μM); ^c^ Not determined; ^d^ SI (Selectivity Index) = IC_50_ (COX-1)/IC_50_ (COX-2).

## Supplementary Materials

The ^1^H-NMR, ^13^C-NMR, DEPT-135, ^1^H-^1^H COSY, HSQC, and HMBC spectra of **1** and **2** are available as supplementary materials available online.
