# Supplementary material for: Chemical Constituents from the Flower of Hosta plantaginea with Cyclooxygenases Inhibition and Antioxidant Activities and Their Chemotaxonomic Significance
Source: Molecules. 2017 Oct 26;22(11):1825. doi: 10.3390/molecules22111825 (PMC6150378; doi:10.3390/molecules22111825)
Supplement: Supplementary file 1 [file molecules-22-01825-s001.pdf]

Supplementary Data

List of Supplementary data

For compound **1**

Figure S1-1. ^{1}H NMR spectrum of **1** in DMSO-d_6………………………… 2

Figure S1-2. ^{13}C NMR spectrum of **1** in DMSO-d_6………………………… 2

Figure S1-3. DEPT 135 spectrum of **1** in DMSO-d_6……………………….. 3

Figure S1-4. HSQC spectrum of **1** in DMSO-d_6…………………………… 3

Figure S1-5. ^{1}H-^{1}H COSY spectrum of **1** in DMSO-d_6……………………. 4

Figure S1-6. HMBC spectrum of **1** in DMSO-d_6…………………………... 4

For compound **2**

Figure S2-1. ^{1}H NMR spectrum of **2** in DMSO-d_6………………………… 5

Figure S2-2. ^{13}C NMR spectrum of **2** in DMSO-d_6………………………… 5

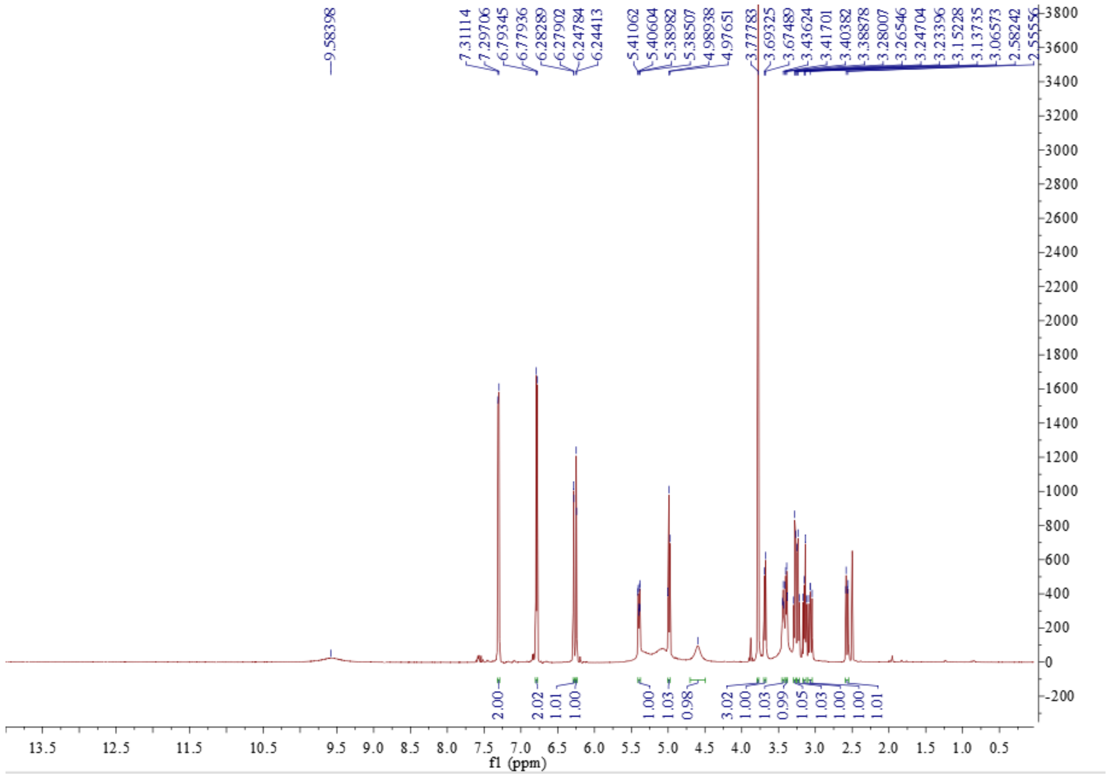

Figure S1-1. ^{1}H NMR spectrum of **1** in DMSO-d_6.

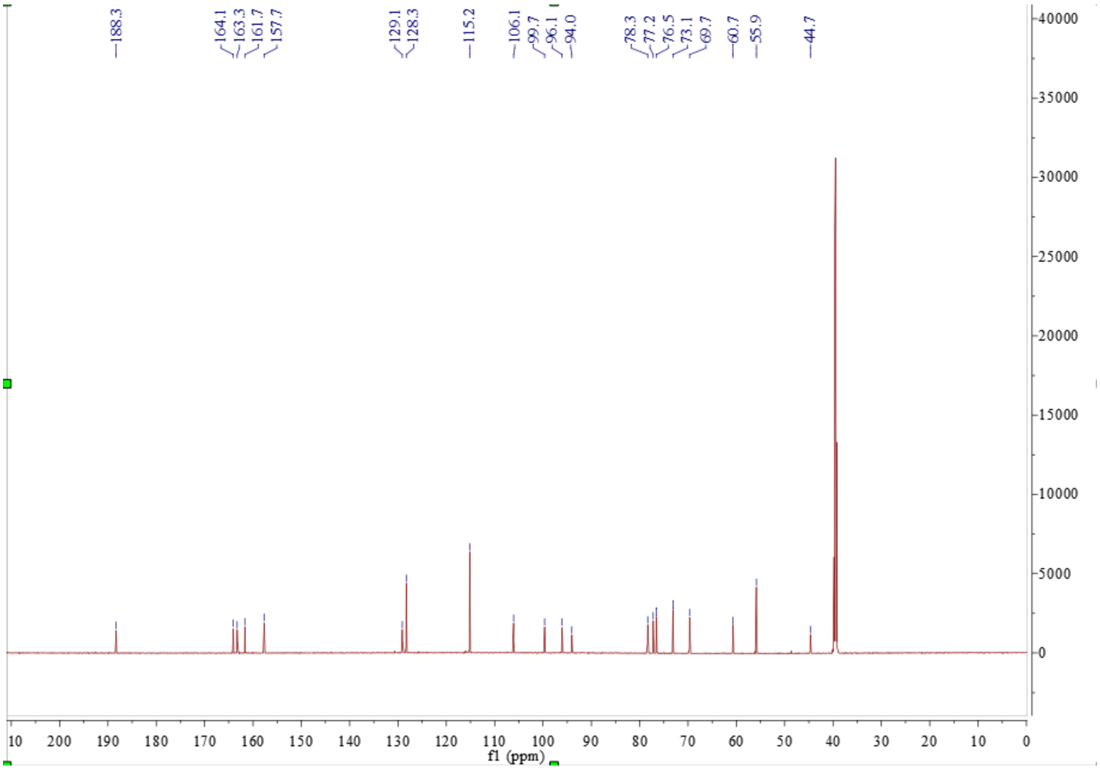

Figure S1-2. ^{13}C NMR spectrum of **1** in DMSO-d_6.

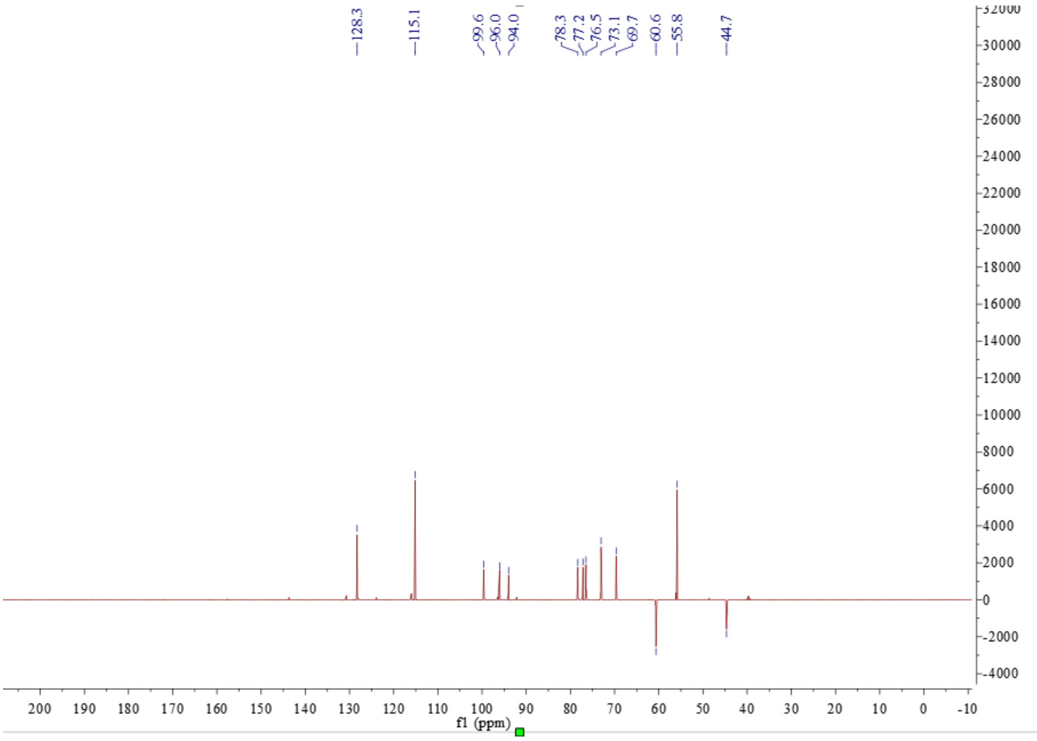

Figure S1-3. DEPT 135 spectrum of **1** in DMSO-d_6.

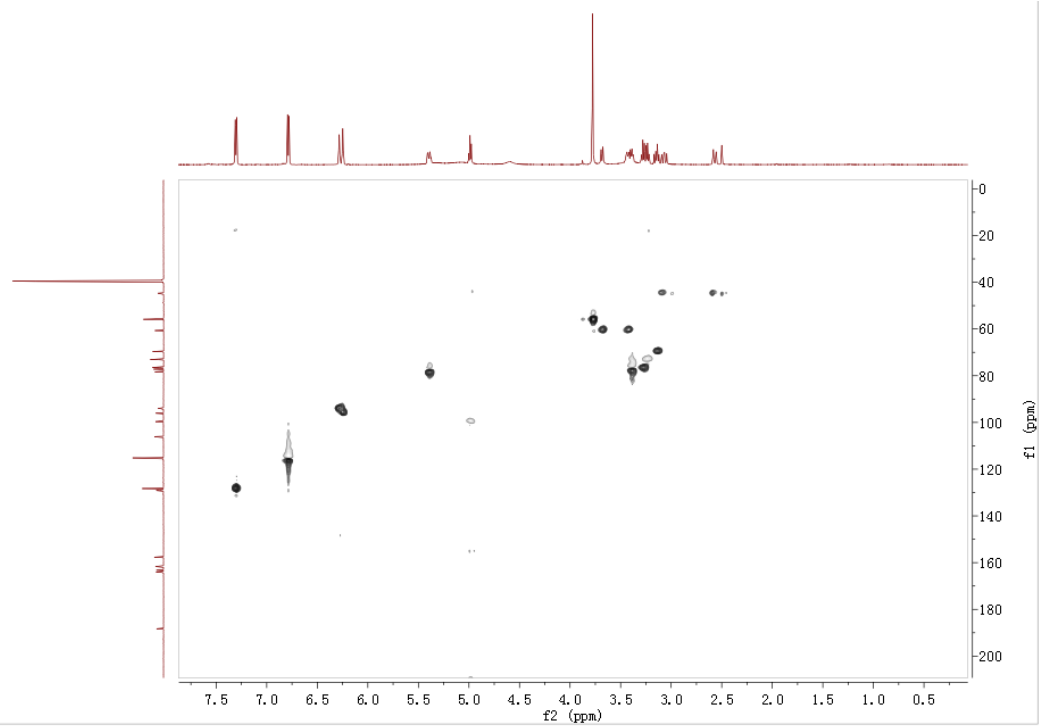

Figure S1-4. HSQC spectrum of **1** in DMSO-d_6.

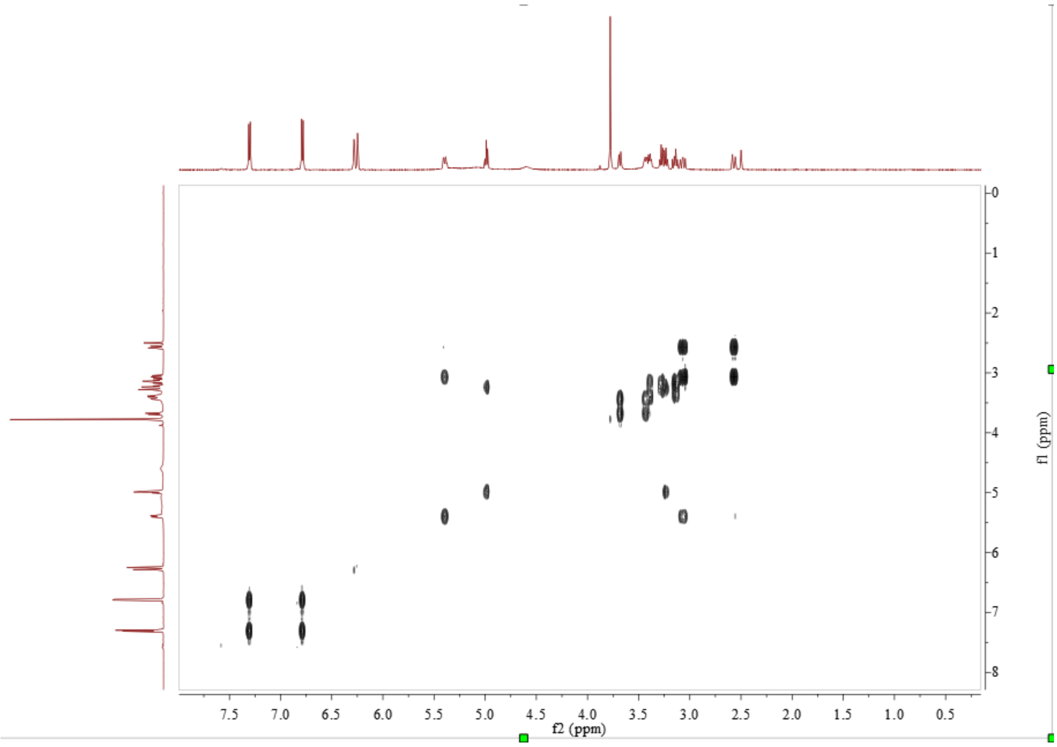

Figure S1-5. ^{1}H-^{1}H COSY spectrum of **1** in DMSO-d_6.

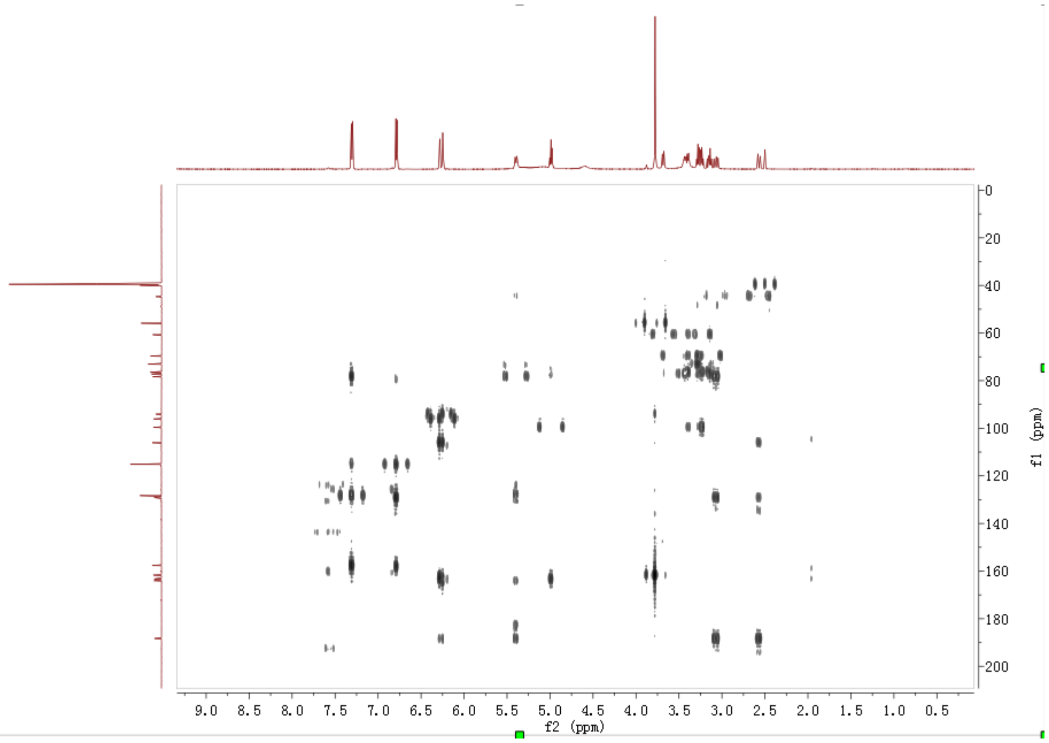

Figure S1-6. HMBC spectrum of **1** in DMSO-d_6.

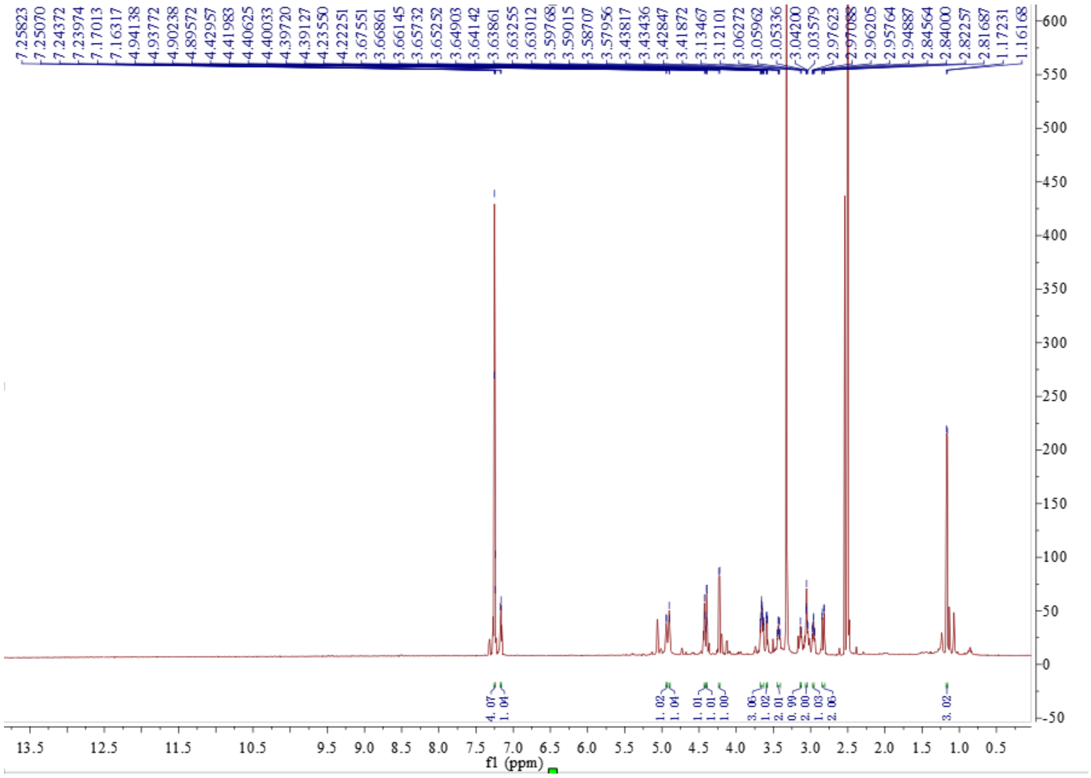

Figure S2-1. ^{1}H NMR spectrum of **2** in DMSO-d_6.

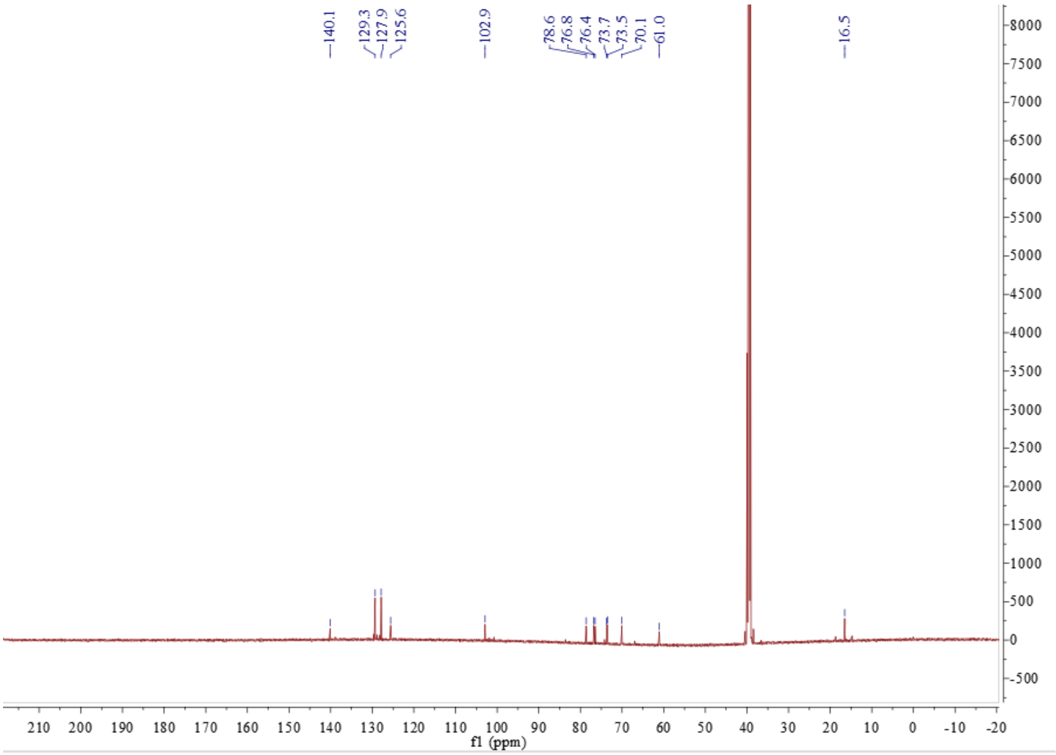

Figure S2-2. ^{13}C NMR spectrum of **2** in DMSO-d_6.
